# A Comparison of Cardiovascular Health Across Migration Flows on the US-Mexico Border

**DOI:** 10.1007/s10903-026-01870-7

**Published:** 2026-03-05

**Authors:** Félice Lê-Scherban, Saishi Cui, Camila A. Picchio, M. Gudelia Rangel, Leah Bakely, J. Eduardo Gonzalez-Fagoaga, Ahmed A. Asadi-Gonzalez, Ana P. Martinez-Donate

**Affiliations:** 1 Department of Epidemiology & Biostatistics, Dornsife School of Public Health, Drexel University, Philadelphia, USA; 2 Drexel Urban Health Collaborative, Dornsife School of Public Health, Drexel University, Philadelphia, USA; 3 Department of Community Health & Prevention, Dornsife School of Public Health, Drexel University, Philadelphia, USA; 4 Mexico Section of the U.S.-Mexico Border Health Commission, Tijuana, Mexico; 5 El Colegio de la Frontera Norte, Tijuana, Mexico; 6 Universidad Autónoma de Baja California, Tijuana, Mexico

**Keywords:** Immigrants, Migrants, Cardiovascular health, Surveys, Mexico-US border

## Abstract

Our objective was to characterize cardiovascular health in different migrant flows crossing through the United States (US)-Mexico border region. Participants (*n* = 1,264; weighted *n* = 682,130) in a probability-based study of migrant flows completed cross-sectional surveys and measurements from February–December 2022 at transit points in Tijuana, Matamoros, and Ciudad Juárez, Mexico. Outcomes were American Heart Association cardiovascular health (CVH) metrics (diet, physical activity, nicotine exposure, body mass index, blood lipids, blood glucose, blood pressure) (all range 0–100). We used covariate-adjusted linear regression to compare CVH outcomes by migration flow (Pre-migration, Return, Southbound, Deported). Compared to Pre-migration participants, Return and Southbound participants had significantly worse cardiovascular health scores for body mass index in adjusted models (difference = -24.0 [-38.9, -9.1] and difference = -14.7 [-26.2, -3.3], respectively), while Deported participants had significantly lower scores for blood lipids (difference = -12.3 [-23.7, -0.9]). We identified two cardiovascular risk factors that may serve as targets for intervention to improve cardiovascular health among migrants on the US-Mexico border. Findings demonstrate that migration flows represent subpopulations whose health needs and barriers to health services differ. Transit points may be potential intervention venues to improve migrant health.

**Supplementary Information:**

The online version contains supplementary material available at https://doi.org/10.1007/s10903-026-01870-7.

## Background

Heart disease is the leading cause of mortality among the nearly 11 million Mexican migrants residing in the United States (US) [1]. Mexican migrants in the US exhibit higher prevalence of cardiovascular disease (CVD) risk factors compared to their US-born counterparts [2], coupled with lower rates of risk factor awareness, treatment, and management [3]. Despite improved healthcare access among long-term residents [4], an extended duration of residence in the US [4] is correlated with a deteriorating CVD risk profile, characterized by elevated levels of obesity, hypertension [5], diabetes [6], and metabolic-associated liver disease [7].

Prior to arrival to the US, many Latino migrants travel through the US-Mexico border region, a pivotal juncture where cultures, economies, and health risks intersect [8]. The journey across the border, often charged with challenges and uncertainties, compounds the risk factors associated with CVD among migrants. Poor access to healthy foods, poor exercise habits [9], stress, and behaviors related to fear of deportation among undocumented migrants can further compound their risk once they are in the US [10]. Moreover, due to patterns of circular migration or deportation, migrants may have CVD or CVD risk factors that require long-term management, impacting their time in both Mexico and the US [11]. In Mexico, CVD represents the leading cause of mortality, accounting for about 20% of total mortality [12]. This disease burden also carries high public monetary cost: roughly 70% of diabetes, obesity, and CVD expenditures in the country are absorbed by three of its public health institutions [12]. The complex interplay of socioeconomic and structural barriers migrants from Mexico experience while in the US, coupled with and contributing to unhealthy lifestyle practices, increases this mobile population’s risk for poor CVD outcomes. However, the comprehensive cardiovascular risk profile of individuals at various stages of the migration process remains understudied [13]. Thus, the objective of this manuscript is to characterize the cardiovascular health profile of different migrant flow populations crossing through the US-Mexico border region.

## Theoretical Framework

This analysis was informed by Zimmerman’s Migration Framework, which accounts for the often circular nature of migration and defines the migration process as a series of phases: pre-departure, transit, destination, interception/deportation, and return [13]. Each phase is characterized by distinct health risks and opportunities for intervention, and not all migrants experience every phase (e.g., interception). In keeping with this framework, our central hypothesis was that cardiovascular health profiles, and by extension needs and opportunities for intervention, differ between Mexican migrants at different migration phases.

### Methods

#### Participants

**Fig. 1:**
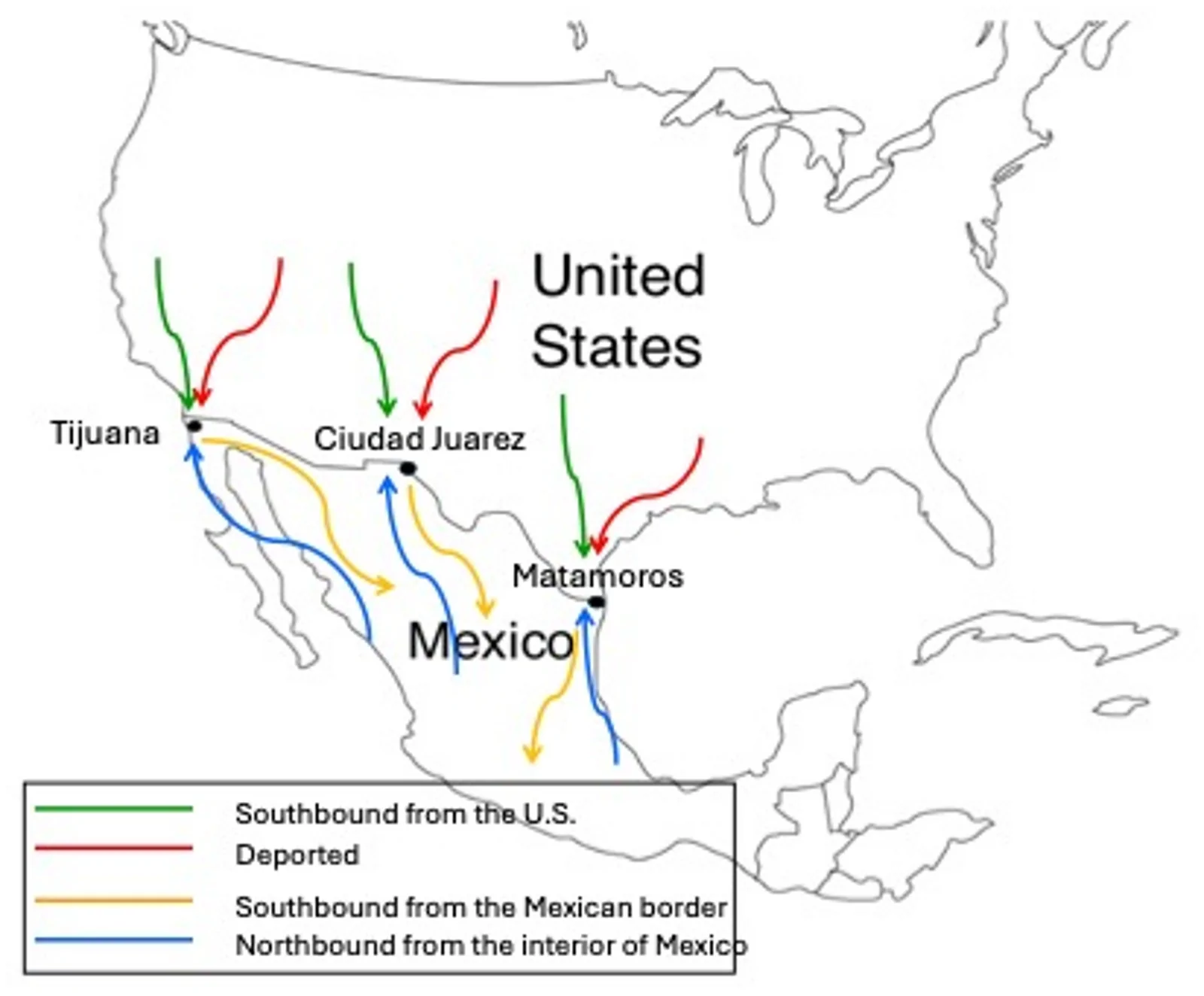
Project Migrante data collection sites and sampled migration flows

The Migrante Project was a binational initiative that conducted health surveys with migrant flows in the US-Mexico border region from 2009 to 2024 [14]. Surveys were conducted at key transit points in Tijuana, Matamoros, and Ciudad Juárez, Mexico. These cities are significant as they receive flows from various sending and receiving regions in the US, Mexico, and other Latin American countries. The study sampled three migrant flows in each city: Deported migrants, Northbound migrants, and Southbound migrants, each representing distinct migration phases and trajectories (Fig. 1).

The Deported Flow comprised migrants released at the Mexican border after deportation from the US and processing by Mexican migration authorities. Migrants in this flow may have been living in the US prior to their detention or could have been intercepted as they were trying to cross to the US. This group is considered highly vulnerable and is a hard-to-reach, understudied migrant population. The Northbound Flow comprised migrants traveling from their communities of origin in Mexico and/or Central America to the border region. This group included new migrants and returnees whose health is influenced by their previous migration history and recent exposures in their home communities. The Southbound Flow comprised migrants returning to their sending communities elsewhere in Mexico or Latin America after a stay in the US or the Mexican border region [14].

The surveys employed a two-stage, probability sampling design at key crossing points such as airports, central bus stations, and deportation stations. Based on a period of continuous enumeration and screening at each sampling site and point within the site (e.g., doorway, ticket desk), a stratified, random sample of “space-time” pairs (space = data collection points, time = month and time of day) for data collection was generated, with probability of selection generally proportional to migrant flow volume. Within each data collection shift, individuals were consecutively approached and screened for eligibility and participation; the total number of adults crossing through the sampling point was also enumerated. Eligible individuals were aged 18 years or older, fluent in Spanish, and not residents of the study cities. Detailed methodology of the Migrante Project is available elsewhere [14]. The project was reviewed and approved by the Institutional Review Board of Drexel University. All participants provided informed consent to participate.

Data for this study originated from a Migrante Project survey focused on non-communicable chronic disease conducted February-December 2022. Out of 1,264 eligible participants, we excluded 10 (0.8%) with missing information for all outcomes. This resulted in an analytic sample of 1,254 participants, representing a weighted population of 678,223: 300 (weighted population: 240,949) who were sampled and participated as part of the Northbound Flow, 626 (weighted population: 395,668) in the Southbound Flow, and 328 (weighted population: 41,605) in the Deported Flow. In keeping with our theoretical framework, we categorized participants in the Northbound Flow as either Pre-migration (*n* = 194, weighted population: 147,538) or Return (*n* = 106, weighted population: 93,412). These categories corresponded to those who reported never having moved to the US to live and those who had previously moved to the US to live, respectively.

#### Data Collection

Data collection used standardized protocols and included health metrics like blood lipids, glucose, and blood pressure, as well as lifestyle factors such as diet, physical activity, and tobacco use. Measurements included height and weight using a portable balance. Blood lipids were assessed using the portable analyzer CardioChekTM (Polymer Technology Systems Diagnostics, Inc., Indianapolis, IN) [15]. Glycohemoglobin (HbA1c) was ascertained with the A1CNow+ portable analyzer (Polymer Technology Systems Diagnostics, Inc., Indianapolis, IN), using a finger stick whole blood rapid test [16]. A portable digital blood pressure monitor (Welch Allyn’s Connex^®^ ProBPTM 3400) was used to assess systolic and diastolic blood pressure levels [17].

Participants were queried about lifestyle factors through questions adapted from the 2020 California Health Interview Survey (CHIS) (diet) and 2018 Mexico National Survey on Health and Nutrition (ENSANUT, per its Spanish acronym) (physical activity and tobacco). To assess diet, participants were asked how frequently they consumed vegetables (excluding fried potatoes and cooked dried beans), fruit (excluding juice), and soda or other sweetened beverages during the past 30 days. To assess physical activity, participants were asked separate questions assessing frequency and duration of moderate and vigorous physical activity in the past 30 days. Nicotine exposure was assessed through questions about lifetime and current cigarette and electronic cigarette use.

#### Measures

The outcome was cardiovascular health (CVH), a construct developed by the American Heart Association (AHA) in 2010 [18] and updated in 2022 [19]. The 2022 iteration incorporates eight components of cardiovascular health (termed “Life’s Essential 8” by the AHA): diet, physical activity, nicotine exposure, sleep health, body mass index (BMI), blood lipids, blood glucose, and blood pressure. The AHA provides specific definitions and cutoffs for each (Table 1) [19]. All measures are expressed on a scale ranging from 0 to 100, with higher scores representing better cardiovascular health. Because information on sleep health was not available in the Migrante Project survey, we did not include this metric in analyses. Additionally, we modified the definitions of the diet and nicotine exposure metrics based on data available in the Migrante Project survey (Table 1). The remaining metrics were defined using the AHA definitions, including alternate scoring for participants aged < 20 years (35 participants; 2.8% of sample). We also calculated an overall CVH score for each participant, defined following AHA guidelines as the unweighted average of the 7 included component metrics [19].

**Table 1:** Definitions of cardiovascular health metrics in the Migrante project and the American heart association’s (AHA’s) “Life’s essential 8” [19]

Metric	Migrante Project definition			AHA definition
Diet	Sum of points for consumption of vegetables, fruit, and sugar-sweetened beverages (SSBs) Scoring for vegetable consumption:			Scoring based on quantiles of Dietary Approaches to Stop Hypertension (DASH) [20]-style diet adherence, such as measured through the Mediterranean Eating Pattern for Americans (MEPA) [21] screener
	**Points**	**Frequency**		
	35 25 10 0	4+ times per day 2-3 times per day 1 time per day < 1 time per day		
	Scoring for fruit consumption:			
	**Points**	**Frequency**		
	35 25 10 0	4+ times per day 2-3 times per day 1 time per day < 1 time per day		
	Scoring for SSB consumption:			
	**Points**	**Frequency**		
	30 25 10 0	Never < 1 time per week 1+ time per week but < 1 time per day 1+ times per day		
Physical activity	Minutes of moderate or vigorous activity per week			Same
	Scoring:			
	**Points**	**Minutes (age 20+)**	**Minutes (age <20)**	
	100 90 80 60 40 20 0	≥ 150 120-149 90-119 60-89 30-59 1-29 0	≥ 420 360-419 300-359 240-299 120-239 1-119 0	
Nicotine exposure	Smoking tobacco or use of inhaled nicotine delivery system (NDS)			Includes additional categories for former smokers who quit 1–5 vs. 5 + years ago, subtracts points for living with an active indoor smoker
	Scoring:			
	**Points**	**Status**		
	100 65 25 0	Never smoker Former smoker, quit ≥ 1 year ago Former smoker, quit < 1year ago or current NDS user Current smoker		
Sleep health	Not available			Self-reported average hours of sleep per night
BMI	BMI (kg/m^2^) Scoring:			Same
	**Points**	**Level (age 20+)**	**Level (age <20)**	
	100 70 30 15 0	<25 25.0-29.9 30.0-34.9 35.0-39.9 ≥40.0	5th-<85th percentile 85th-<95th Percentile 95th percentile-<120% of the 95th Percentile 120% of the 95th percentile-<140% of the 95th percentile ≥140% of the 95th percentile	
Blood lipids	Non-HDL cholesterol (mg/dL) Scoring:			Same
	**Points**	**Level (age 20+)**	**Level (age <20)**	
	100 60 40 20 0 If drug treated, subtract 20 points	<130 130-159 160-189 190-219 ≥220	<100 100-119 120-144 145-189 ≥190	
Blood glucose	HbA1c (%) Scoring:			Same
	**Points**	**Level**		
	100 60 30 20 10 0	No history of diabetes, <5.7 No history of diabetes, 5.7-6.4 Diabetes, <7.0 Diabetes, 8.0-8.9 Diabetes, 9.0-9.9 Diabetes, ≥10.0		
Blood pressure	Systolic and diastolic blood pressure (SBP and DBP) (mm Hg)			Same
	Scoring:			
	**Points**	**Level**		
	100 75 50 25 0 If drug treated, subtract 20 points	<120 SBP/<80 DBP 120-129 SBP/<80 DBP 130-139 SBP or 80-89 DBP 140-159 SBP or 90-99 DBP ≥160 SBP or 2≥100 DBP		

Covariates were chosen a priori based on known associations with migration status and cardiovascular health: age (years), gender (male/female), health insurance status during the previous 12 months (insured all the time/insured never or sometimes/unknown), education (less than high school/high school/more than high school), and marital status (married or cohabiting/other status). For descriptive purposes, participants were categorized as having a history of US migration if they reported that their country of residence was the US, if they had ever migrated to the US to live, or if they had spent at least 30 days in the US during the previous 12 months.

#### Analysis

We computed descriptive statistics for sample characteristics and CVH metric scores. The significance of differences for continuous variables was assessed using ANOVA, and for categorical variables using the chi-square test. Associations of migration flow with CVH scores (each CVH metric and overall CVH score) were examined separately using unadjusted and covariate-adjusted weighted linear regression models. Analyses incorporated survey weights accounting for the complex survey design and participation [14]. Because data missingness was limited almost exclusively to the outcomes (CVH metrics), we used a complete-case approach for analysis [22], using all available observations for each outcome.

Three sensitivity analyses were conducted to test robustness of results. First, to address possible confounding by prior history of CVD or CVD treatment, a sensitivity analysis was performed in which models were further adjusted for participant-reported history of CVD (diagnosis of heart attack, angina pectoris, heart failure, or embolism/stroke) and clinically diagnosed CVD risk factors (hypertension, diabetes, or cholesterolemia). Second, to test the influence of large survey weights on results, analyses were repeated after trimming the survey weights based on the methodology described by Liu et al. [23]. A third sensitivity analysis tested models additionally adjusted for lifetime spent in the US (categorized as < 1 year, 1-4.9 years, 5-9.9 years, or 10 + years), which may be conceptualized as a possible mediator; the cross-sectional data precluded causal mediation analysis.

## Results

Overall, combining all migration phases, participants had a mean age of 44.9 years and were predominantly male (62%) (Table 2). 55% had a high school degree or higher education, approximately half (51%) were married or cohabiting, 55% reported having health insurance during the entire previous 12 months, and 48% had a history of previous migration to the US. Participants in the Deported phase were, on average, younger, more likely to be male, and had lower education levels compared to participants in the other phases (Table 2). There were also differences in health insurance status by migration flow, with 88% of those in the Deported phase and 75% in the Return phase reporting having had insurance never or only sometimes during the previous 12 months, compared to 30% among Southbound and 46% among Pre-migration participants. There were no statistically significant differences in cardiovascular risk or disease history by migration phase, with 18% of the total population reporting having clinically diagnosed hypertension, diabetes, or cholesterolemia and 0.7% reporting a history of diagnosed cardiovascular disease.

**Table 2:** Sample characteristics, by migration phase

	Unweighted *n*						Weighted % or mean					
	Total	Pre-migration	Return	South-bound	Deported		Total	Pre-migration	Return	South-bound	Deported	
	*N* = 1,**254**	*n* = 194	*n* = 106	*n* = 626	*n* = 328		*N* = 678,**223**	*n* = 147,**538**	*n* = 93,**412**	*n* = 395,**668**	*n* = 41,**605**	
	*n*	*n*	*n*	*n*	*n*		% or mean	% or mean	% or mean	% or mean	% or mean	*p* ^a^
Age in years (n, mean)	1,254	194	106	626	328		44.9	41.0	43.8	48.0	33.2	< 0.001
Male sex (vs. female) (n, %)	870	122	69	385	288		62.2	67.5	71.5	55.4	88.7	< 0.001
*Education level (n*,* %)*												
Less than high school	682	113	50	287	229		45.5	62.1	34.3	40.0	67.4	< 0.001
High school	332	42	29	178	82		28.1	22.5	36.6	28.1	28.5	
Bachelor’s degree or higher	250	39	27	161	17		26.4	15.3	29.1	31.9	4.2	
*Marital status (n*,* %)*												
Married or cohabiting	674	95	51	358	165		51.2	50.6	50.5	51.1	59.9	0.25
Single	535	85	52	240	154		38.3	39.8	49.1	35.0	35.9	
Other	55	14	3	28	9		10.4	10.0	0.4	13.9	4.2	
*Health insurance during past 12 months (n*,* %)*												
All the time	491	93	33	358	7		55.1	54.8	25.0	69.0	0.6	< 0.001
Never or sometimes	708	100	73	260	275		43.0	46.2	75.0	30.0	88.0	
Missing/unknown	65	1	0	8	46		1.9	0.0	0.0	1.1	11.5	
Ever migrated to the US to live (n, %)	745	0	106	363	269		48.3	0.0	100.0	51.1	73.6	< 0.001
*Total years spent in US (n*,* %)*												< 0.001
< 1 (includes none)	772	194	32	343	194		63.5	100.0	28.6	57.7	64.4	< 0.001
1-4.99	100	0	13	71	16		6.7	0.0	4.6	9.9	3.2	
5-9.99	64	0	7	34	16		3.0	0.0	6.0	3.0	7.8	
10 or more	303	0	53	172	77		24.2	0.0	49.0	27.8	21.4	
Missing	25	0	1	6	18		2.7	0.0	11.8	1.6	3.1	
History of cardiovascular disease (n, %)^b^	8	1	1	6	0		0.7	0.0	4.0	1.2	0.0	0.33
History of clinical cardiovascular risk factor (n, %)^c^	192	35	30	83	44		18.0	20.4	25.6	15.3	20.6	0.59

^a^Calculated using ANOVA for continuous variables, chi-square test for categorical variables

^b^Diagnosis of heart attack, angina pectoris, heart failure, and/or embolism/stroke

^c^Diagnosis or medication for hypertension, diabetes, and/or cholesterolemia

Available sample size differed between metrics (Table 3). Mean scores also varied substantially between metrics, ranging from 31.6 (SD 23.6) for diet to 90.7 (SD 20.9) for blood lipids (Table 3). Mean total CVH score was 64.5 (SD 16.3) when incorporating all nonmissing information and 65.9 (SD 12.4) when including only information for participants with nonmissing information for all seven metrics. Mean BMI scores varied by migration phase (*p* = .001), ranging from 47.0 (SD 25.7) among Return to 70.5 (SD 30.0) among Pre-migration participants. There were no statistically significant differences or consistent patterns in the other CVH metrics or CVH score by migration flow.

**Table 3:** Cardiovascular health (CVH) metric scores, by migration phase

	Unweighted *n*						Weighted mean (SD)					
	Total	Pre-migration	Return	South-bound	Deported		Total	Pre-migration	Return	South-bound	Deported	
Metric^a^	*n*	*n*		*n*	*n*		mean (SD)	mean (SD)	mean (SD)	mean (SD)	mean (SD)	*p* ^c^
Diet score	1,052	178	97	535	242		31.6 (23.6)	29.5 (24.0)	27.7 (20.4)	34.2 (24.3)	23.6 (19.4)	0.10
Physical activity score	1,235	193	103	616	323		58.7 (45.8)	61.1 (45.2)	63.2 (45.3)	57.6 (45.8)	51.4 (47.9)	0.56
Nicotine exposure score	1,251	194	106	623	328		84.7 (33.1)	84.9 (35.6)	88.8 (27.0)	84.0 (33.2)	81.2 (35.9)	0.74
Body mass index score	902	150	60	458	234		56.1 (31.8)	70.5 (30.0)	47.0 (25.7)	51.9 (32.0)	65.1 (25.9)	0.001
Blood lipids score	767	129	57	389	192		90.7 (20.9)	93.2 (15.5)	90.7 (20.8)	90.9 (21.3)	80.0 (28.7)	0.14
Blood glucose score	942	140	71	470	261		75.6 (30.8)	75.8 (27.8)	75.1 (27.4)	74.6 (32.8)	83.9 (25.6)	0.21
Blood pressure score	1,054	174	74	526	280		63.9 (31.8)	65.2 (31.3)	70.0 (30.8)	61.7 (32.2)	70.2 (30.2)	0.27
Total CVH score, all available^b^	1,254	194	106	626	328		64.5 (16.3)	66.4 (16.7)	64.4 (12.1)	63.9 (16.9)	64.2 (16.6)	0.83
Total CVH score, nonmissing only^b^	478	90	33	246	109		65.9 (12.4)	68.2 (11.7)	63.5 (11.4)	65.1 (12.8)	67.4 (10.6)	0.53

^a^All metrics are scores ranging 0-100, with higher scores denoting better cardiovascular health

^b^Calculated for each individual as the average component CVH metric scores. “All available” includes all available metrics. “Nonmissing only” includes only participants with nonmissing scores for all 7 included metrics

^c^Calculated using ANOVA

**Fig. 2:**
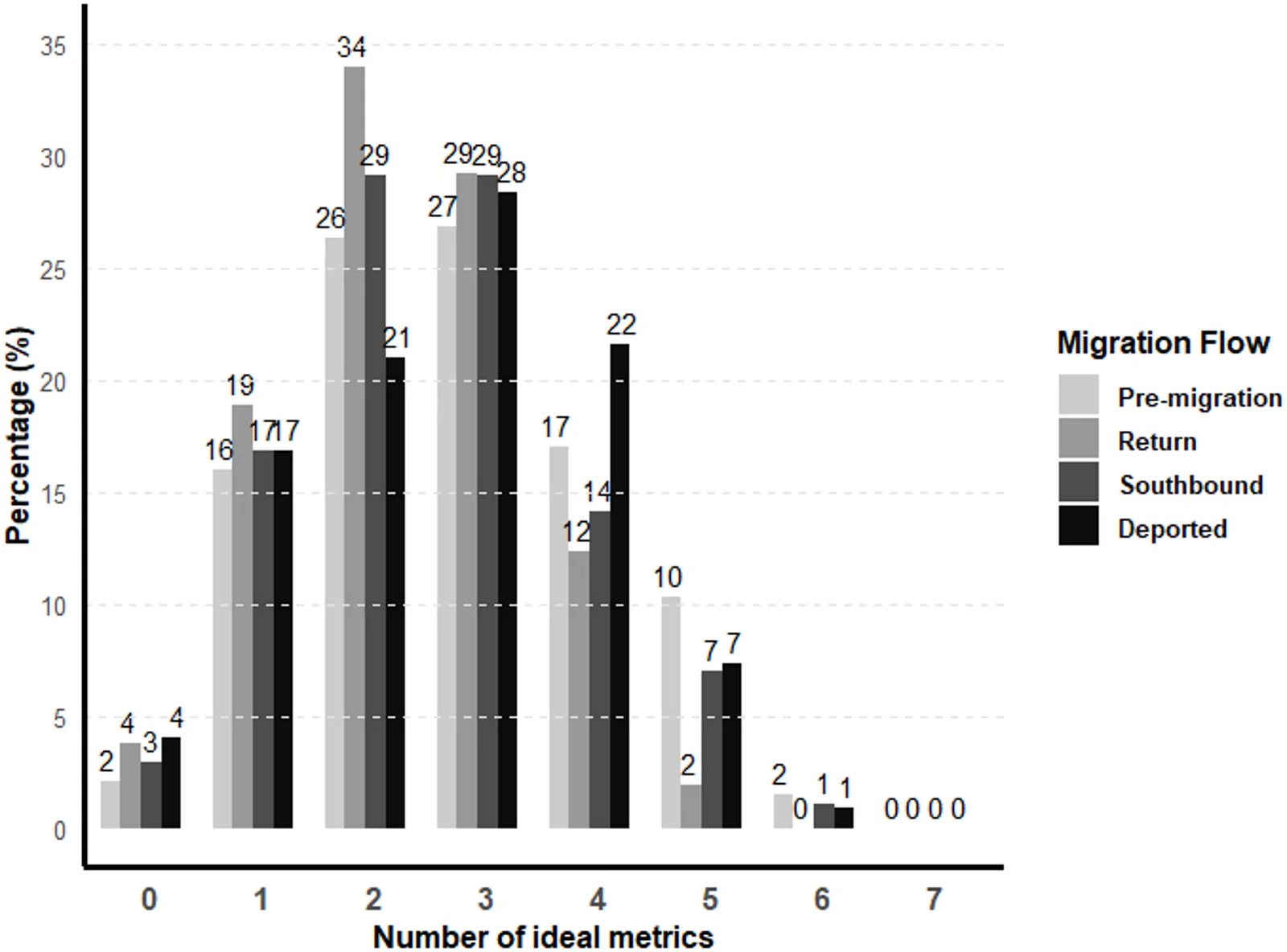
Distribution of number of ideal CVH metrics (i.e., score = 100), by migration phase. Figure includes 478 participants (90 Pre-migration, 33 Return, 246 Southbound, 109 Deported) with nonmissing information for all 7 metrics

Among participants with nonmissing information for all seven metrics, none had ideal status (i.e., score = 100) for all metrics (Fig. 2). Participants in the Return phase were more likely to have few ideal metrics: 57% had only 0–2 ideal metrics compared to 42% among Deported, 44% among Pre-migration, and 49% among Southbound.

In adjusted linear regression models, compared to Pre-migration participants, mean scores for BMI were lower (denoting worse cardiovascular health) among both Return (difference = -24.0 [95% confidence interval − 38.9, -9.1]) and Southbound (difference = -14.7 [-26.2, -3.3]) participants (Table 4). Compared to Pre-migration participants, mean score for blood lipids was lower among participants in the Deported phase (difference = -12.3 [-23.7, -0.9]). Of note, Southbound participants had lower adjusted mean scores for every metric except blood glucose, as well as lower overall total CVH score, compared to Pre-migration participants. However, differences ranged in magnitude and were statistically significant only for body mass index. Results for Return and Deported participants were less consistent in direction. Results from sensitivity analyses were very similar to those from the main analysis with the exception that in adjusted models using trimmed weights, blood lipids scores were no longer lower among Deported compared to Pre-migration participants (see Supplement).

**Table 4:** Associations of migration phase with cardiovascular health (CVH) metric scores from weighted linear regression models

			Unadjusted				Adjusted^b^	
		Return (vs. Pre-immigration)	Southbound (vs. Pre-immigration)	Deported (vs. Pre-immigration)		Return (vs. Pre-immigration)	Southbound (vs. Pre-immigration)	Deported (vs. Pre-immigration)
	*n*	Diff (95% CI)	Diff (95% CI)	Diff (95% CI)		Diff (95% CI)	Diff (95% CI)	Diff (95% CI)
Diet score	1,052	-1.7 (-13.5, 10.0)	4.8 (-6.8, 16.4)	-5.9 (-16.3, 4.5)		-3.1 (-12.5, 6.4)	-0.2 (-8.6, 8.2)	-0.2 (-8.4, 7.9)
Physical activity score	1,235	2.1 (-18.5, 22.7)	-3.5 (-18.5, 11.5)	-9.7 (-26.3, 7.0)		1.6 (-17.5, 20.6)	-0.1 (-13.0, 12.8)	-11.4 (-28.4, 5.6)
Nicotine exposure score	1,251	4.0 (-11.5, 19.4)	-0.9 (-12.3, 10.6)	-3.7 (-16.5, 9.1)		4.0 (-9.9, 17.8)	-4.7 (-15.1, 5.7)	2.2 (-12.6, 16.9)
Body mass index score	902	**-23.5 (-38.2**,** -8.7)**	**-18.6 (-31.4**,** -5.8)**	-5.4, (-18.2, 7.5)		**-24.0 (-38.9**,** -9.1)**	**-14.7 (-26.2**,** -3.3)**	-8.8 (-22.9, 5.2)
Blood lipids score	767	-2.5 (-16.7, 11.8)	-2.2 (-12.0, 7.6)	**-13.2 (-25.8**,** -0.6)**		-0.1 (-12.2, 12.1)	-1.8 (-11.3, 7.7)	**-12.3 (-23.7**,** -0.9)**
Blood glucose score	942	-0.7 (-14.9, 13.4)	-1.2 (-13.5, 11.1)	8.1 (-3.9, 20.1)		3.1 (-11.3, 17.4)	1.1 (-8.9, 11.1)	4.7 (-7.1, 16.5)
Blood pressure score	1,054	4.5 (-10.2, 19.1)	-3.5 (-14.2, 7.2)	4.9 (-6.8, 16.6)		7.9 (-5.9, 21.7)	-1.4 (-10.4, 7.6)	0.7 (-10.7, 12.1)
Total CVH score ^a^	1,254	-2.0 (-7.9, 3.9)	-2.5 (-7.8, 2.8)	-2.1 (-7.8, 3.6)		-1.6 (-8.2, 4.9)	-1.8 (-6.7, 3.1)	-1.3 (-7.4, 4.8)

Bold indicates *p*<.05

^a^Total score is average of individual metric scores. Includes participants with missing information for 1 or more metrics

^b^Adjusted for age, gender, health insurance, education, and marital status

## Discussion

In a 2022 survey at transit points in three Mexican cities with large migrant flows on the Mexico-US border, the mean overall CVH score on a scale from 0 (worst) to 100 (best) was 64.5, indicating moderate CVH [24]. This is similar to data from the general US adult population where an analysis using national US data from the 2013–2018 National Health and Nutrition Examination Survey (NHANES) found a mean CVH score (calculated using all 8 CVH metrics) of 64.7 [24]. Mean scores were also similar in the Migrante Project and NHANES populations for many of the individual metrics, although they were higher (indicating better cardiovascular health) in the Migrante Project population for blood lipids and nicotine exposure, and higher in NHANES for diet, especially among women. The low levels of diagnosed cardiovascular disease (< 1%) and CVD risk factors (18%) in the Migrante Project are a stark contrast to the estimated 48% and 37% of adult population who have a CVD in the US [25] and Mexico [26], respectively. These differences might suggest a healthier CVD profile among migrants included in this study. However, they may also reflect modifications of the CVD metrics in the Migrante Project definitions compared to NHANES (Table 1) or the specific Spanish-language terminology used in the questions. For example, participants may have underreported prior diagnoses if the terms used in the questions differed from those used by their healthcare providers when they received the diagnoses. This may be particularly relevant for those who may have received a diagnosis in the US, as well as undiagnosed disease or risk factors due to limited healthcare access in the migrant population.

Scores for nearly all CVH outcomes were lower among Southbound than Pre-migration individuals after adjustment for demographic differences, although most differences were small and interpretation was limited by imprecise estimates. Differences in CVH metric scores among Return and Deported compared to Pre-migration flows were less consistent in direction but were statistically significantly worse for BMI among Return and blood lipids among Deported participants. While the cross-sectional data preclude causal conclusions, these differences may to some extent reflect higher exposure to unhealthier lifestyles and poor access to healthcare in the US among Southbound, Return, and Deported participants. This is consistent with prior research, including in the Migrante Project, linking residence in the US to declines in cardiovascular health indicators among immigrants [27–30]. Another potential contributor to worse health among Southbound and Return participants is “salmon bias,” a hypothesized phenomenon in which immigrants who are in poor health are more likely to return to their sending country [31]. However, previous research in the Migrante Project and other datasets has found limited evidence of salmon bias for cardiovascular health [31]. Finally, changes over time in health selection, in which healthier individuals are more likely to migrate—also known as the ‘healthy migrant effect’—may have played a role in explaining the relatively better cardiovascular profile of the Pre-migration flow. Ro and Fleischer found evidence of increasing health selection over time with respect to obesity among Mexican immigrants to the US [32]. However, an analysis of an earlier wave of Migrante Project data found little evidence of health selection [33].

More generally, patterns of migration between the US and Mexico are complex and often characterized by circular migration, complicating interpretation of differences between migration flows. Future research accounting for this complexity is necessary to fully understand how cardiovascular risk develops over time among migrants. Prior research has found worse health and healthcare among Mexican migrants with circular migration patterns compared to non-migrants and migrants who remain the US [30]. In the current sample, Return-phase participants were the least likely to report having health insurance during the entire previous year. The robustness of our results to additional adjustment for time spent in the US suggests a role of mechanisms beyond duration of US residence that are specific to the experiences of each migration flow.

Regardless of mechanisms, our results highlight the need for services to meet the health needs of migrants with complex migration experiences. This includes ensuring consistent access to and coordination of healthcare on both sides of the US-Mexico border to reduce migration-related disruptions. In a prior Migrante Project survey, rates of healthcare utilization were below those in other studies of adults in both the US and Mexico [34]. Migrants also experience low levels of health insurance access: in the current sample, only 55% of participants across all migrant flows reported being continuously insured during the past 12 months. Limited access to healthcare services at all phases of the migration experience may lead to undiagnosed as well as untreated cardiovascular risk factors and disease, which in turn can result in more severe disease levels and the need for more expensive treatment services.

Interpretation of results from this study is limited by the cross-sectional data. In addition, the need to sample separately among multiple different flows and locations resulted in relatively small sample size per migration flow, limiting the precision of estimates and precluding examination of differences among migrants from different sending communities and countries within each flow. There was substantial item missingness for some outcome metrics, particularly those reliant on clinical measurement. Overall CVH scores were slightly higher when including only participants with nonmissing information for all 7 metrics (Table 3), suggesting that scores for metrics with high missingness may be biased upward. Although surveys were collected at three different border-crossing hubs, results may not be generalizable to migrants crossing the border at other locations. Migrants speaking languages other than Spanish were also excluded. Future research may shed additional light by including children and youth, including unaccompanied minors.

## New Contribution to the Literature

Our analysis found that overall cardiovascular health among migrants on the US-Mexico border was moderate. Scores for individual metrics were lowest for diet, physical activity, and BMI, suggesting these may be targets for intervention to improve cardiovascular health among this population. Existing CVD was rare at the time of survey (< 1%), suggesting opportunities for effective primary prevention. Findings also underscore that migration flows represent subpopulations whose health needs and barriers to health services on both sides of the border differ. More generally, the Migrante Project has demonstrated that transit locations at border-crossing hubs can serve as effective interception points to collect needed information about health and healthcare needs among migrants on the US-Mexico border, and offer rapid tests for CVD risk factors. Next steps may test these interception points as venues for interventions to improve migrant health.

## Supplementary Information

Below is the link to the electronic supplementary material.

Supplementary Material 1

## Data Availability

Migrante Project data are available from the Inter-University Consortium of Political and Social Research at https://www.icpsr.umich.edu/web/DSDR/studies/38601/datadocumentation.
