# Supplementary material for: A Comparison of Cardiovascular Health Across Migration Flows on the US-Mexico Border
Source: J Immigr Minor Health. 2026 Mar 5;28(5):1134–45. doi: 10.1007/s10903-026-01870-7 (PMC13371472; doi:10.1007/s10903-026-01870-7)
Supplement: Supplementary file 1 [file 10903_2026_1870_MOESM1_ESM.docx]

**Supplement**

# Additional adjustment for participant-reported history of cardiovascular disease (CVD) and clinically diagnosed CVD risk factors

Results were nearly identical after additional adjustment for history of CVD and having a previously diagnosed CVD risk factor.

Table S1. Associations of migration phase with cardiovascular health (CVH) metric scores from weighted linear regression models

|  |  | **Adjusted^b^** | | |
| --- | --- | --- | --- | --- |
|  |  | **Return**  **(vs. Pre-migration)** | **Southbound**  **(vs. Pre-migration)** | **Deported**  **(vs. Pre-migration)** |
|  | **n** | **Diff (95% CI)** | **Diff (95% CI)** | **Diff (95% CI)** |
| Diet score | 1052 | -3.1 (-12.4, 6.2) | 0.2 (-7.6, 8.0) | -0.5 (-8.9, 8.0) |
| Physical activity score | 1235 | 1.3 (-17.6, 20.3) | 0.1 (-12.9, 13.1) | -11.8 (-29.0, 5.4) |
| Nicotine exposure score | 1251 | 3.9 (-10.1, 17.8) | -4.2 (-14.5, 6.1) | 1.7 (-13.1, 16.5) |
| Body mass index score | 902 | **-24.3 (-39.1, -9.5)** | **-13.9 (-25.2, -2.6)** | -9.5 (-23.3, 4.3) |
| Blood lipids score | 767 | -0.1 (-12.1, 11.9) | -1.9 (-10.8, 7.0) | -12.0 (-24.1, 0.1) |
| Blood glucose score | 942 | 3.3 (-9.8, 16.4) | -0.9 (-10.0, 8.1) | 7.6 (-3.9, 19.0) |
| Blood pressure score | 1054 | 8.3 (-5.4, 22.0) | -2.5 (-11.4, 6.5) | 2.3 (-9.1, 13.6) |
| Total CVH score ^a^ | 1254 | -1.5 (-8.1, 5.0) | -1.9 (-6.7, 3.0) | -1.1 (-7.2, 5.1) |
| **Bold** indicates p<.05 |  |  |  |  |
| ^a^ Total score is average of individual metric scores. Includes participants with missing information for 1 or more metrics. | | | | |
| ^b^ Adjusted for age, gender, health insurance, education, marital status, history of CVD, and diagnosed CVD risk factor. History of CVD is defined as having received a diagnosis of heart attack, angina pectoris, heart failure, and/or embolism/stroke. History of diagnosed CVD risk factor is defined as having received a diagnosis or medication for hypertension, diabetes, and/or cholesterolemia. | | | | |

# Trimming survey weights

Weights were trimmed using the method described by Liu et al.^1^

Table S3. Distribution of survey weights before and after trimming

|  | **Untrimmed** | **Trimmed** |
| --- | --- | --- |
| N | 1254 | 1254 |
| Weights trimmed (n (%)) | --- | 307 (24%) |
| Range (min–max) | 2.8–23,991.1 | 2.8–2110.4 |
| Median | 116.4 | 86.5 |
| Mean | 540.8 | 333.4 |

Results from models incorporating the trimmed weights were similar to those from the main analysis except that blood lipids scores were not lower among participants in the Deported than Pre-migration phase.

Table S4. Associations of migration stage with CVH scores from weighted linear regression models incorporating trimmed survey weights

|  |  | **Adjusted^b^** | | |
| --- | --- | --- | --- | --- |
|  |  | **Return**  **(vs. Pre-migration)** | **Southbound**  **(vs. Pre-migration)** | **Deported**  **(vs. Pre-migration)** |
|  | **n** | **Diff (95% CI)** | **Diff (95% CI)** | **Diff (95% CI)** |
| Diet score | 1052 | 1.0 (-5.6, 7.7) | 1.8 (-3.0, 6.5) | -1.7 (-7.3, 3.9) |
| Physical activity score | 1235 | -1.1 (-14.2, 12.0) | 0.2 (-9.4, 9.7) | -8.2 (-19.5, 3.0) |
| Nicotine exposure score | 1251 | 6.7 (-1.4, 14.8) | -3.3 (-11.5, 5.0) | 0.4 (-9.4, 10.2) |
| Body mass index score | 902 | **-15.3 (-26.0, -4.6)** | **-8.7 (-16.8, -0.6)** | 0.0 (-8.6, 8.7) |
| Blood lipids score | 767 | -1.6 (-8.1, 5.0) | -5.3 (-11.3, 0.7) | 0.2 (-5.8, 6.3) |
| Blood glucose score | 942 | 1.4 (-8.8, 11.7) | -4.5 (-12.3, 3.4) | 6.5 (-1.8, 14.8) |
| Blood pressure score | 1054 | 2.0 (-8.3, 12.4) | 1.4 (-5.7, 8.4) | 3.7 (-3.9, 11.3) |
| Total CVH score ^a^ | 1254 | 0.8 (-3.4, 4.9) | -1.3 (-5.0, 2.4) | 1.2 (-3.0, 5.4) |
| **Bold** indicates p<.05 |  |  |  |  |
| ^a^ Total score is average of individual metric scores. Includes participants with missing information for 1 or more metrics. | | | | |
| ^b^ Adjusted for age, gender, health insurance, education, and marital status. | | | | |

# Additional adjustment for lifetime time spent in the US

Results were similar after additional adjustment for lifetime time spent in the US.

Table S2. Associations of migration phase with cardiovascular health (CVH) metric scores from weighted linear regression models

|  |  | **Adjusted^b^** | | |
| --- | --- | --- | --- | --- |
|  |  | **Return**  **(vs. Pre-migration)** | **Southbound**  **(vs. Pre-migration)** | **Deported**  **(vs. Pre-migration)** |
|  | **n** | **Diff (95% CI)** | **Diff (95% CI)** | **Diff (95% CI)** |
| Diet score | 1052 | 1.8 (-11.2, 14.8) | 2.3 (-8.0, 12.5) | 3.8 (-8.5, 16.1) |
| Physical activity score | 1235 | -5.5 (-27.7, 16.7) | -3.9 (-18.0, 10.3) | -13.9 (-32.1, 4.3) |
| Nicotine exposure score | 1251 | -0.8 (-15.1, 13.4) | -7.5 (-18.8, 3.8) | 0.1 (-15.2, 15.5) |
| Body mass index score | 902 | **-25.9 (-43.5, -8.3)** | **-17.0 (-29.3, -4.6)** | -7.1 (-22.4, 8.2) |
| Blood lipids score | 767 | -0.3 (-12.7, 12.1) | -2.2 (-12.4, 8.0) | **-13.3 (-24.4, -2.1)** |
| Blood glucose score | 942 | 8.5 (-7.4, 24.3) | 4.8 (-6.0, 15.7) | 5.9 (-6.3, 18.2) |
| Blood pressure score | 1054 | 10.8 (-5.2, 26.8) | 0.1 (-10.1, 10.3) | 1.3 (-10.6, 13.3) |
| Total CVH score ^a^ | 1254 | -2.5 (-10.2, 5.1) | -2.6 (-7.8, 2.5) | -2.3 (-8.5, 4.0) |
| **Bold** indicates p<.05 |  |  |  |  |
| ^a^ Total score is average of individual metric scores. Includes participants with missing information for 1 or more metrics. | | | | |
| ^b^ Adjusted for age, gender, health insurance, education, marital status, and lifetime time spent in the US (<1 year, 1-4.9 years, 5-9.9 years, 10+ years). | | | | |

# References

1.	Liu B, Ferraro D, Wilson E, Brick JM. Trimming extreme weights in household surveys. Paper presented at: American Statistical Association Section on Survey Research Methods;2004; Toronto.
